# Identification of potential key variants in mandibular premolar hypodontia through whole-exome sequencing

**DOI:** 10.3389/fgene.2023.1248326

**Published:** 2023-09-08

**Authors:** Shinyeop Lee, Hyunsoo Ahn, Hyeonhye Kim, Kwanghwan Lee, Sanguk Kim, Jae Hoon Lee

**Affiliations:** ^1^ Department of Prosthodontics, College of Dentistry, Yonsei University, Seoul, Republic of Korea; ^2^ Graduate School of Artificial Intelligence, Pohang University of Science and Technology, Pohang, Republic of Korea; ^3^ Tufts University School of Medicine, Boston, MA, United States; ^4^ Department of Life Sciences, Pohang University of Science and Technology, Pohang, Republic of Korea

**Keywords:** hypodontia, genotype-phenotype correlation, genetic association studies, bioinformatics, extracellular matrix

## Abstract

Determining genotype–phenotype correlations in patients with hypodontia is important for understanding disease pathogenesis, although only a few studies have elucidated it. We aimed to identify genetic variants linked to non-syndromic bilateral mandibular second premolar hypodontia in a Korean population for the first time by specifying the phenotype of hypodontia. Twenty unrelated individuals with non-syndromic bilateral mandibular second premolar hypodontia were enrolled for whole-exome sequencing. Using a tooth agenesis gene set panel consisting of 112 genes based on literature, potential candidate variants were screened through variant filtering and prioritization. We identified 13 candidate variants in 12 genes, including a stop-gain variant (c.4750C>T) in *LAMA3*. Through the functional enrichment analysis of the prioritized genes, several terms related to tooth development were enriched in a protein–protein interaction network of candidate genes for mandibular premolar hypodontia. The hypodontia group also had approximately 2-fold as many mutated variants in all four genes related to these key terms, which are *CDH1*, *ITGB4*, *LAMA3*, *LAMB3*, as those in the 100 healthy control group individuals. The relationship between enriched terms and pathways and mandibular premolar hypodontia was also investigated. In addition, we identified some known oligodontia variants in patients with hypodontia, strengthening the possibility of synergistic effects in other genes. This genetic investigation may be a worthwhile preliminary attempt to reveal the pathogenesis of tooth agenesis and sets a background for future studies.

## 1 Introduction

Congenital tooth agenesis, which is a failure in tooth development, is the most common dental anomaly in humans (De Coster et al., 2009). Tooth agenesis can alter masticatory efficiency, aesthetic appearance, and speech. Studies have found that significant facial changes can occur with congenital tooth agenesis (Taju et al., 2018). Since teeth tend to migrate towards edentulous regions, missing teeth may result in poor positioning of the remaining teeth, making it difficult to manage proper oral rehabilitation. Early diagnosis of tooth agenesis is crucial so that long-term treatment plans, such as the conservation of deciduous teeth or the maintenance of appropriate space for lost teeth for dental implants, can be established for the patients (Nunn et al., 2003).

Different terms describe tooth agenesis based on the number of missing teeth, excluding third molars. When less than six teeth fail to develop, tooth agenesis is referred to as “hypodontia.” When six or more teeth are missing, tooth agenesis is called “oligodontia,” and “anodontia” refers to the developmental failure of all teeth. Hypodontia is associated with approximately 150 genetic syndromes, including cleft lip and/or cleft palate (Vieira, 2003); however, non-syndromic tooth agenesis (NSTA), in which only dentition is affected as familial or sporadic forms, can also occur without systemic genetic syndromes. Hypodontia can be caused by environmental factors, such as infection, trauma, or early radiation therapy, but is mainly associated with genetic mutations (Ye and Attaie, 2016).

The overall prevalence of hypodontia is 6.4%, but this varies within different continents and racial groups (Khalaf et al., 2014). Mandibular second premolars are the most commonly affected teeth, and there are no specific studies on the prevalence of bilateral mandibular second premolar hypodontia. However, we can assume the occurrence to be approximately 1% from a meta-analysis report, considering that approximately 21% of tooth agenesis patients have missing bilateral mandibular premolars, and approximately 67% of patients with more than one missing tooth have two missing teeth (Polder et al., 2004).

Teeth develop via a series of complex signaling interactions between the oral epithelium and neural crest-derived mesenchyme through epithelial thickening (initiation) and bud, cap, and bell stages (Tucker and Sharpe, 2004). These molecular signaling pathways are under strict genetic control during development (Yin and Bian, 2015). Numerous factors, such as nuclear factor (NF)-κB, wingless-related integration site (Wnt), bone morphogenic protein (Bmp), fibroblast growth factor (FGF), and sonic hedgehog (Shh) families, are considerably involved in the regulation of tooth development. Any alteration in these factors can potentially contribute to hypodontia (Galluccio et al., 2012). Since mutations in the *MSX1* and *PAX9* were found to cause NSTA (Vastardis et al., 1996; Stockton et al., 2000), more than 18 genes have been identified as additional causes (AlFawaz et al., 2015; Bonczek et al., 2017; Williams and Letra, 2018). A recent systematic review aimed to investigate the NSTA phenotypes associated with gene mutations and revealed patterns of tooth agenesis based on the identity of mutated genes. For example, mutations in *MSX1* and *PITX2* were reported to be responsible for premolar and third molar agenesis, whereas *PAX9* mutations resulted in agenesis in all molars and mandibular central incisors (Fournier et al., 2018). Two *WNT10A* variants cause maxillary lateral incisor agenesis (Mostowska et al., 2015). In addition, rs15705 and rs3178250 variants of *BMP2* increase the risk of mandibular incisor agenesis (Lu et al., 2016). Despite accumulating evidence for different hypodontia phenotype pathogeneses, research in this field is very limited. Hypodontia shows variable expressivity due to the numerous ways in which 28 unique, permanent teeth could be absent or positioned. Additionally, mutations in several genes contribute to diverse phenotypes with the possibility of oligogenic inheritance. A study that performed genotype–phenotype analysis in patients with *WNT10A* mutations reported that *WNT10A* mutations were associated with the number and type of tooth agenesis, such as biallelic *WNT10A* mutations associated with absence of maxillary and mandibular molars as well as mandibular central incisors. However, one limitation of this study was the candidate gene analysis (Arzoo et al., 2014).

Recent developments in next-generation sequencing have enabled rapid and efficient analysis of personal DNA genome sequences. Whole-exome sequencing (WES) has received particular attention for the identification of genetic variants of diseases. Exomes refer to all exons within the genome and represent approximately 1.5% of the human genome. WES has been proven effective because up to 90%–85% of known disease-related variants are contained in exons. Earlier, traditional research on non-syndromic hypodontia focused on finding variants in candidate genes, but current trends using WES have revealed new mutated genes for non-syndromic hypodontia (Zeng et al., 2017; Dinckan et al., 2018; Zhang et al., 2019), including *OPN3*, which was previously thought to be irrelevant to tooth development (Inagaki et al., 2021). Genotype–phenotype correlation in hypodontia is important not only for understanding the pathogenesis of the disease but also for the specific tooth regeneration required for replacement eventually (Chhabra et al., 2014; Williams and Letra, 2018). By specifying the phenotype of non-syndromic hypodontia as agenesis of the bilateral mandibular second premolar and applying WES and bioinformatics in the Korean population, in this study, we aimed to better understand the underlying mechanisms of hypodontia by identifying commonalities between hypodontia-causing variants.

## 2 Materials and methods

### 2.1 Participants

Twenty unrelated individuals were included in the study. The participants were selected based on the following criteria: congenital absence of bilateral mandibular second premolars, presence of all other permanent teeth (excluding the third molars), and absence of any other genetic syndromes. They were examined by one dentist for case eligibility using radiographic and clinical examinations and were excluded if they showed any other craniofacial anomaly or syndrome. Eligibility was confirmed after asking participants about their dental and medical histories, including pedigree data, to assess their family history. Panoramic radiographic images, clinical records, and written informed consent were obtained from all patients in this study. One hundred healthy exome sequencing data from 3,703 Ansan–Ansung cohort participants provided by the National Biobank of Korea, the Korea Disease Control and Prevention Agency, Republic of Korea (KBN-2021-063) were used as controls. This clinical study was approved by the Institutional Review Board of the Armed Forces Medical Command (AFMC-20085-IRB-20-085; approved on 3 November 2020). This clinical study was conducted in accordance with the Declaration of Helsinki.

### 2.2 DNA sample collection

A 2-mL saliva sample was obtained from each participant using an Oragene DNA self-collection kit (OG-500; DNA Genotek). Each saliva sample was mixed with a solution from the collection kit, which stabilized the sample at room temperature. Genomic DNA was extracted using a preplT-L2P DNA extraction kit (DNA Genotek) according to the manufacturer’s instructions. DNA Link inc. performed DNA collection, extraction, and whole-exome sequencing. The concentration and purity of the DNA samples were evaluated using agarose gel electrophoresis and a PicoGreen dsDNA Assay (Invitrogen).

### 2.3 Whole-exome sequencing (WES)

Briefly, exons were captured according to the manufacturer’s instructions using SureSelect Human All Exon V5 (Agilent Technologies), and 2 × 100 base pair reads were sequenced using an Illumina Novaseq 6,000 system (Illumina). FASTQC v0.11.5 was used to assess the quality of the read sequences. Each read was compared to the Genome Reference Consortium Human Build 37, and the Burrows–Wheeler Aligner package v0.7.12 was used to align the reads with the reference genome. The reads were realigned around indels, and base quality was recalibrated based on changes using GenomeAnalysisTK (GATK) v3.5. After these steps, alignment quality control was performed, and the variants were called using the GATK HaplotypeCaller gVCF mode. Raw variants were recalibrated using GATK v3.5, and finally, analysis-ready VCF files were produced using filtered variants.

### 2.4 Filtering of exomic variants and data analysis

We applied two different approaches. First, Fisher’s exact test was performed to compare the allele frequencies of each variant between the case-control groups. Odds ratios with 95% confidence intervals were used to evaluate the corresponding risks of variants and mandibular premolar hypodontia. PLINK v1.9 was used as the statistical software. Variants with an association *p* < 1 × 10^−7^ and odds ratios >1 were considered statistically significant. We then applied an additional filter with variants annotated as MODERATE or HIGH impact on SnpEff v 4.2 (Cingolani et al., 2012). Finally, we selected variants with a total allele frequency (AF) < 0.01 in the genome aggregation database (gnomAD) v2.1.1. GnomAD has the largest population variation collection in databases and is used to identify pathogenic variants (Karczewski et al., 2020). Data are available on the gnomAD website (https://gnomad.broadinstitute.org/) (Gudmundsson et al., 2022).

Second, we constructed a gene set panel for previously identified genes involved in syndromic tooth agenesis (STA) or NSTA, consisting of 112 genes from literature (Yin and Bian, 2015; Williams and Letra, 2018; Andersson et al., 2020). Whole gene names are listed in Supplementary Table S1. From the genetic variants in the gene set panel, we selected variants annotated as MODERATE or HIGH impact on SnpEff v 4.2 with total AFs under 0.01 in both the 1000G project and gnomAD v2.1.1.

### 2.5 Prioritization of mutated variants

To prioritize and elucidate the pathogenicity of mutated variants that may cause mandibular premolar hypodontia, we applied 18 *in silico* prediction tools: Mutation Taster, PROVEAN, SIFT, SIFT4G, Polyphen2, MetaSVM, REVEL, LIST-S2, LRT, M-CAP, Mutation Assessor, BayesDel noAF, MetaLR, DANN, EIGEN, EIGEN PC, FATHMM, and FATHMM-MKL. SnpEff was used to identify missense variants from SIFT and PolyPhen2. In addition, the scores of Combined Annotation-Dependent Depletion (CADD) were checked for candidate variants. CADD integrates multiple annotations, such as sequence conservation score and ENCODE project functional annotations, and expresses the variants’ deleteriousness as a number (Kircher et al., 2014). The CADD score can be considered a ranking, and the higher the score, the more deleterious the variant. We prioritized variants that were estimated as deleterious by at least half of the *in silico* prediction tools (more than medium in the case of Mutation Assessor) and had CADD scores above 20. Prioritized variants were compared with the Human Gene Mutation Database to check if they were previously reported in tooth agenesis. Prioritized variants were finally ruled out if they were classified as benign using the ClinVar database or the American College of Medical Genetics and Genomics (ACMG) guidelines. According to a recent report, Sanger sequencing validation was not performed, considering we only detected single nucleotide variants, and the coverage depth of WES obviated the need (Arteche-López et al., 2021).

### 2.6 *In silico* functional analysis of candidate genes related to the disease

We applied the computational tool STRING v11.5 (Szklarczyk et al., 2019) to examine the pathways and functional terms associated with the candidate genes of mandibular premolar hypodontia. We inputted 12 candidate genes obtained from WES with “*homo sapiens*” as the organism. We used the multiple proteins query and selected “full STRING network,” “medium confidence level,” and “medium false discovery rate (FDR) stringency.” A protein–protein interaction (PPI) network was visualized, and functional enrichment analysis was performed in the network. The following categories were analyzed for functional enrichment using Fisher’s exact test with Benjamini–Hochberg FDR correction: Gene Ontology (GO), STRING Clusters, KEGG Pathways, Reactome Pathways, Wiki Pathways, Disease-gene associations (DISEASES), Subcellular localization (COMPARTMENTS), Human Phenotype (Monarch), UniProt Annotated Keywords, and SMART protein domains. To compare the total number of mutations in the candidate genes between the case-control groups, the alternate/reference allele ratio of all variants’ sites was calculated. Both synonymous and non-synonymous variants were obtained and odds ratios with *p*-values were performed by Fisher’s exact test and Bonferroni’s correction.

## 3 Results

### 3.1 Patient characteristics

**FIGURE 1:**
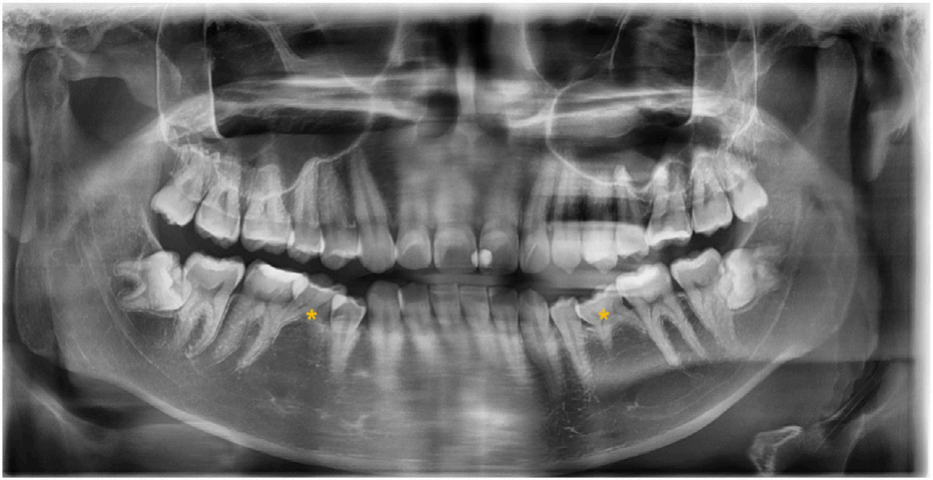
Panoramic radiograph of the subject with a congenital lack of mandibular second premolars (indicated with asterisks).

The 20 samples for WES analysis comprised 18 males and 2 females. The median age of the participants in the sample was 21 years (range: 19–65 years). After clinical and radiographic evaluations, all patients were diagnosed with non-syndromic bilateral mandibular second premolar hypodontia (Figure 1). No other signs of genetic syndromes were found in any of the patients.

### 3.2 Candidate variants found in WES analysis and prioritization

A total of 112,487 variants were found in the 20 samples after performing WES. Of these, 853 variants were considered statistically significant after the case-control group comparison, but none met the prioritization criteria. From the gene set panel, 13 variants in 12 genes were found in 11 patients. Of these, 12 variants were missense, and 1 variant was stop-gain, and all 13 variants were heterozygous. No variant was identified in the tooth agenesis gene set panel from the other nine patients. We divided these variants into three categories: known variants, candidate variants in NSTA genes, and candidate variants in STA genes.

### 3.3 Known variants

Two variants from three patients were identified from samples previously reported in oligodontia patients (Table 1). A known missense variant (c.1138A>C) in *EDAR* was found in two patients (Zeng et al., 2017). This variant has been reported pathogenic for non-syndromic oligodontia but benign for ectodermal dysplasia in the ClinVar database. Another known missense variant (c.511C>T) in *WNT10A* was also found in these samples (Song et al., 2014; Zeng et al., 2017; Park et al., 2019; Zhao et al., 2019; Zhou et al., 2021; Xie et al., 2022).

**TABLE 1:** List of known variants.

Variant							*In silico* pathogenicity prediction																		CADD score
Patient no.	Effect	Gene name	HGVS.c	HGVS.p	Transcripts	AF	1	2	3	4	5	6	7	8	9	10	11	12	13	14	15	16	17	18	
1	missense	*EDAR*	c.1138A>C	p.Ser380Arg	NM_022336.4	0.001	DC	N	D	D	PD	D	LDC	D	D		N	D	T	U	U	U	D	D	28
2	missense	*EDAR*	c.1138A>C	p.Ser380Arg	NM_022336.4	0.001	DC	N	D	D	PD	D	LDC	D	D		N	D	T	U	U	U	D	D	28
3	missense	*WNT10A*	c.511C>T	p.Arg171Cys	NM_025216.3	0.001	DC	D	D	D	PD	T	LDC	D	N		M	T	T	U	U	U	T	D	24.8

HGVS.c, coding variant; HGVS.p, protein-level variant; Transcripts, mRNA, transcript number; AF, allele frequency from gnomAD v.2 database; *in silico* prediction tools (1-Mutation Taster, 2-PROVEAN, 3-SIFT, 4-SIFT4G, 5-Polyphen2, 6-MetaSVM, 7-REVEL, 8-LIST-S2, 9-LRT, 10-M-CAP, 11-Mutation Assessor, 12-BayesDel noAF, 13-MetaLR, 14-DANN, 15-EIGEN, 16-EIGEN PC, 17-FATHMM, and 18-FATHMM-MKL); DC, disease causing; D, deleterious/damaging; LDC, likely disease causing; PD, probably damaging; U, uncertain significance; T, tolerated; N, neutral; M, medium); CADD, Combined Annotation-Dependent Depletion.

### 3.4 Candidate variants in NSTA genes

Five candidate variants were identified in the five NSTA genes (Table 2). A missense variant (c.697G>A) in *WNT10A* has not been previously reported, but a different nucleotide change in the same codon (c.697G>T) was identified in a previous study (Dinckan et al., 2018). No variant was found in *MSX1, PAX9, AXIN2, PITX2, EDA,* or *EDARRD*.

**TABLE 2:** List of candidate variants in NSTA genes.

Variant							*In silico* pathogenicity prediction																		CADD score
Patient no.	Effect	Gene name	HGVS.c	HGVS.p	Transcripts	AF	1	2	3	4	5	6	7	8	9	10	11	12	13	14	15	16	17	18	
4	missense	*WNT10A*	c.697G>A	c.697G>A	NM_025216.3	0.00007	DC	N	D	D	PD	D	LDC	D	D	D	M	D	D	U	P	P	T	D	29.3
5	missense	*FGFR1*	c.2294G>A	c.2294G>A	NM_023110.3	0.00002	DC	D	D	D	PD	T	LDC	D	D	D	N	D	T	P	U	U	D	D	27.6
6	stop gained	*LAMA3*	c.4750C>T	c.4750C>T	NM_198129.4	0.0001																			36
7	missense	*KREMEN1*	c.196C>A	c.196C>A	NM_001039570.3	0.0005	DC	D	D	D	PD	T	LB	D	D	D	M	T	T	U	P	P	T	D	24.8
8	missense	*SMOC2*	c.947C>T	c.947C>T	NM_001166412.2	0.0002	DC	D	D	D	PD	T	LB	D	D	D	M	T	T	U	U	U	T	D	25.6

HGVS.c, coding variant; HGVS.p, protein level variant; Transcripts, mRNA, transcript number; AF, allele frequency from gnomAD v.2 database; *in silico* prediction tools (1-Mutation Taster, 2-PROVEAN, 3-SIFT, 4-SIFT4G, 5-Polyphen2, 6-MetaSVM, 7-REVEL, 8-LIST-S2, 9-LRT, 10-M-CAP, 11-Mutation Assessor, 12-BayesDel noAF, 13-MetaLR, 14-DANN, 15-EIGEN, 16-EIGEN PC, 17-FATHMM, and 18-FATHMM-MKL); DC, disease causing; D, deleterious/damaging; LDC, likely disease causing; PD, probably damaging; U, uncertain significance; T, tolerated; N, neutral; L, low; M, medium; CADD, Combined Annotation-Dependent Depletion; *, stop gained codon.

### 3.5 Candidate variants in STA genes

Six variants in six STA genes were identified from the samples (Table 3). Patient 9 had a missense variant (c.3052G>A) in *FLNB* with a missense variant (c.329C>A) in *BBS1*. The *FLNB* variant has conflicting interpretations as three submissions as uncertain significance and one as benign, and the *BBS1* variant was of uncertain significance in the ClinVar database. *FLNB* mutation caused abnormal cranium morphology in a mouse model (Lu et al., 2007). Patient 11 had a missense variant (c.2494G>A) in CDH1. A member of the cadherin superfamily, *CDH1* is a transmembrane adhesion protein (Taneyhill, 2008). By regulating cell–cell adhesion or interacting with Wnt intracellular signaling, cadherins serve critical roles in craniofacial morphogenesis and dental development (Schambony et al., 2004; Bienz, 2005; Brembeck et al., 2006; Di Benedetto et al., 2015).

**TABLE 3:** List of candidate variants in STA genes.

Variant							*In silico* pathogenicity prediction																		CADD score
Patient no.	Effect	Gene name	HGVS.c	HGVS.p	Transcripts	AF	1	2	3	4	5	6	7	8	9	10	11	12	13	14	15	16	17	18	
3	missense	*ITGB4*	c.4564C>T	p.Arg1522Cys	NM_000213.5	0.00002	DC	D	D	D	PD	T	U	D	D	D	M	D	T	B	B	B	T	D	24.2
4	missense	*EVC2*	c.1516G>A	p.Glu506Lys	NM_147127.5	0.000003	DC	N	T	D	PD	D	LDC	D	N	D	M	D	D	U	U	U	T	D	25.4
9	missense	*FLNB*	c.3052G>A	p.Val1018Met	NM_001457.4	0.0002	DC	N	T	D	PD	D	LDC	D	D	D	M	D	D	U	U	U	D	D	23.8
9	missense	*BBS1*	c.329C>A	p.Pro110His	NM_024649.5	0.00001	DC	D	D	D	PD	D	LDC	D		D	M	D	D	U	P	P	D	D	25
10	missense	*LAMB3*	c.734G>A	p.Arg245His	NM_000228.3	0.0003	DC	N	D	D	PD	T	LB	D	D	D	M	T	T	P	U	B	T	N	23.9
11	missense	*CDH1*	c.2494G>A	p.Val832Met	NM_004360.5	0.0001	DC	N	D	D	PD	D	LDC	D	D	D	M	D	D	U	P	P	T	D	28.7

HGVS.c, coding variant; HGVS.p, protein level variant; Transcripts, mRNA, transcript number; AF, allele frequency from the gnomAD v.2 database; *in silico* prediction tools (1-Mutation Taster, 2-PROVEAN, 3-SIFT, 4-SIFT4G, 5-Polyphen2, 6-MetaSVM, 7-REVEL, 8-LIST-S2, 9-LRT, 10-M-CAP, 11-Mutation Assessor, 12-BayesDel noAF, 13-MetaLR, 14-DANN, 15-EIGEN, 16-EIGEN PC, 17-FATHMM, and 18-FATHMM-MKL); DC, disease-causing; D, deleterious/damaging; LDC, likely disease-causing; PD, probably damaging; U, uncertain significance; B, benign; T, tolerated; N, neutral; M, medium; CADD, Combined Annotation-Dependent Depletion.

### 3.6 Predicted effect of a stop-gain variant in LAMA3

**FIGURE 2:**
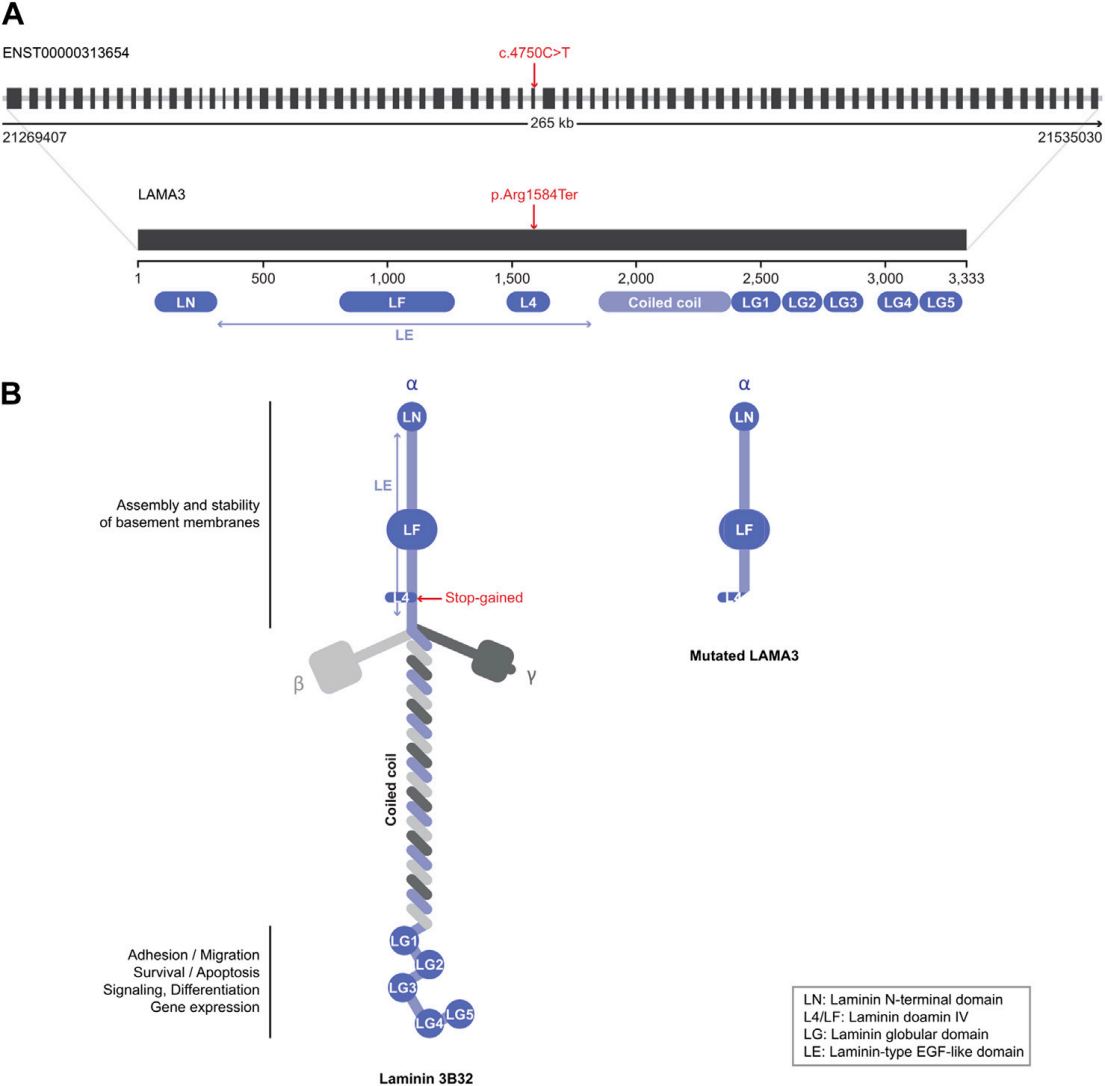
Schematic view of the LAMA3 structure. **(A)** Introns are presented as gray lines and coding exons as black boxes (upper structure). Variants identified in this study are indicated by red characters. **(B)** Native (left) and mutated (right) LAMA3 protein structures. Each domain’s name and role are explained in the figure.

We focused on a stop-gain variant (c.4750C>T) in *LAMA3* found in patient 6. The transcript domain was located in the laminin IV type A domain, causing termination at the protein position 1,584 instead of 3,333 (Figure 2). This variant can lead to functional consequences due to production of a truncated protein or degradation of the transcript by Nonssense-Mediated Decay (NMD). Mutated LAMA3 protein loses coiled-coil and globular laminin domains, which have adhesion, signaling, differentiation, and gene expression functions. The CADD score was very high at 36, and the variant’s clinical significance was conflicting interpretations of pathogenicity with two uncertain significances for junctional epidermolysis bullosa and one likely benign in the ClinVar database (accession number VCV000551372.4).

### 3.7 *In silico* functional study of the candidate genes

**FIGURE 3:**
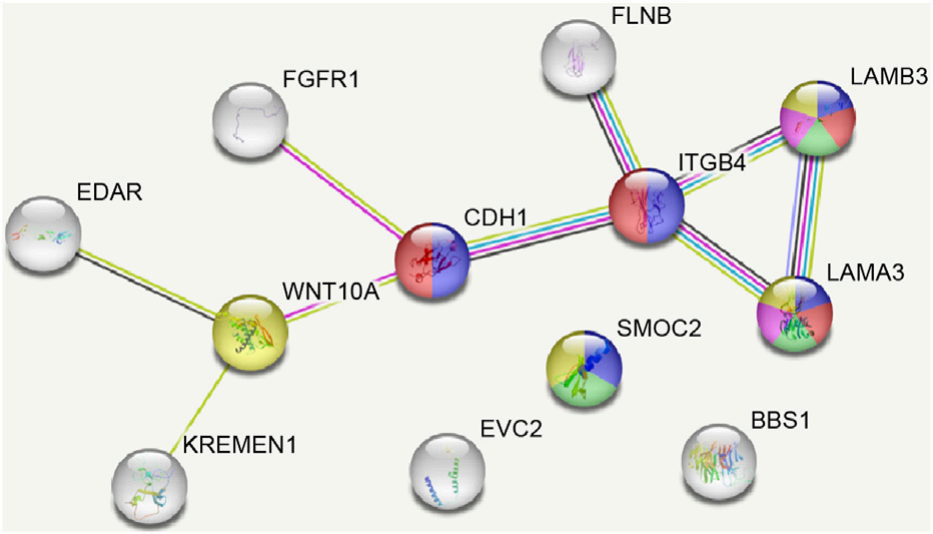
The protein–protein interaction network of variants found in mandibular premolar hypodontia predicted by STRING. Network nodes and edges represent proteins and protein–protein associations, respectively.

The PPI network for our candidate genes for mandibular premolar hypodontia was visualized using STRING (Figure 3). In total, 343 terms were functionally enriched. Of these, 13 were enriched in GO, 8 in KEGG Pathways, 9 in Reactome Pathways, 7 in Wiki Pathways, 12 in DISEASES, 1 in COMPARTMENTS, 280 in Monarch, 12 in UniProt Annotated Keywords, and 1 in SMART. Whole functional enrichment analysis results are shown in Supplementary Table S2. The terms were mainly related to “cell junction,” “extracellular matrix,” “laminin,” and “basement membrane.” We color-coded the key terms in the PPI network, and these are elaborated on in Table 4. Eight out of the 12 candidate genes were found to be significant in case-control comparisons of alternate/reference allele ratio. In particular, hypodontia groups had mutated variants approximately 2-fold as much in all four genes related to “cell junction assembly” and “extracellular matrix organization” (Table 5).

**TABLE 4:** Key functional enrichment analysis result terms.

Color	Category	Term ID	Term description	Observed gene count	Background gene count	Strength	FDR	Gene
red	GO Process	GO:0034329	Cell junction assembly	4	280	1.37	0.028	ITGB4,CDH1,LAMA3,LAMB3
blue	GO Process	GO:0030198	Extracellular matrix organization	5	338	1.38	0.0049	ITGB4,CDH1,LAMA3,SMOC2,LAMB3
green	GO Component	GO:0005604	Basement membrane	3	96	1.71	0.0227	LAMA3,SMOC2,LAMB3
magenta	COMPARTMENTS	GOCC:0005610	laminin-5 complex	2	7	2.67	0.0286	LAMA3,LAMB3
yellow	UniProt Keywords	KW-0272	Extracellular matrix	4	265	1.39	0.0013	WNT10A,LAMA3,SMOC2,LAMB3

The colors are those from Figure 3. FDR; *p*-value after false discovery rate correction.

**TABLE 5:** Fisher’s exact test results of the alternate/reference allele ratio of candidate genes related to key functional terms between the case-control groups.

Gene	Odds ratio	*p*-value	Case allele count		Control allele count	
			Ref	Alt	Ref	Alt
*CDH1*	2.092093	1.35E-44	2,199	721	15,703	2,461
*ITGB4*	2.004838	1.81E-178	6,911	3,129	70,734	15,974
*LAMA3*	1.868188	1.55E-85	6,048	1712	56,560	8,570
*LAMB3*	1.574748	7.63E-30	2,524	1,356	19,589	6,683

## 4 Discussion

To the best of our knowledge, this is the first study to reveal the underlying genetic variants in a specific phenotype of hypodontia using WES and bioinformatics analyses. We selected bilateral mandibular second premolar hypodontia as a phenotype for the following reasons. First, because rodents do not have premolars, it is difficult to unveil the pathogenesis of premolar agenesis using experiments. Second, maxillary and mandibular teeth are reported to be developed by different genetic programs (Ferguson et al., 2000; Cobourne and Sharpe, 2003), and extrinsic factors could be involved in unilateral hypodontia (De Coster et al., 2009).

While a meta-analysis of the prevalence of dental agenesis was conducted (Polder et al., 2004), there may still exist a considerable number of individuals with dental agenesis who are unaware of their condition. Moreover, since there is no specific research for the prevalence of bilateral mandibular second premolar hypodontia, we had to speculate the prevalence from a meta-analysis result. These facts may have affected the outcomes and be a limitation for the study.

No significant variants were found in the case-control group comparisons. We were unable to identify the specific variant that may affect the agenesis of the mandibular second premolars. This might be due to the small sample, but considering the genetic heterogeneity of tooth agenesis, it is probable that a single critical variant for the specific phenotype of the disease may not exist. Instead, considering oligodontia is a rare disease (almost 0.1% prevalence) and a severe form of tooth agenesis, it is interesting that the same variants reported to cause oligodontia were also found in these hypodontia patients. Missense variants (c.1138A>C) and (c.511C>T) in *EDAR* and *WNT10A*, respectively, have been previously reported in oligodontia patients. The results of recent WES investigations in tooth agenesis proposed the idea of mutational load with oligogenic inheritance and multilocus variation models (Posey et al., 2017; Dinckan et al., 2018; Andersson et al., 2020). This variable expressivity reinforces the theory that variations in several genes, working either alone or in concert with other genes, may determine the severity of NSTA (Arte et al., 2013; Dreesen et al., 2014; Williams and Letra, 2018).

Notably, *LAMA3*, *LAMB3*, and *ITGB4* are involved in junctional epidermolysis bullosa (Fine et al., 2008). These proteins are involved in basement membrane-mediated cell adhesion and are critical for proper tooth development (Aberdam et al., 1994; Asaka et al., 2009). Key terms from functional enrichment analysis included “cell junction,” “extracellular matrix,” “basement membrane,” and “laminin.” Moreover, the hypodontia group had approximately 2-fold as many mutated variants in all four genes related to these key terms as the control group. Basement membranes are specialized extracellular matrices composed primarily of different types of laminin, type IV collagen, perlecan, and nidogen (Timpl, 1996). During early development, they act as a physical barrier and regulatory structure for reciprocal signaling between the oral epithelium and mesenchyme layers. These basement membrane matrices regulate proliferation, polarity, and adhesion, as well as the size and morphology of tooth germs (Yoshizaki and Yamada, 2013). Early in the development of the mouse embryo, type IV collagen and laminin molecules are evenly distributed in the dental basement membranes, and they vanish as the membranes are broken down by enzymes (Lesot et al., 1981; Kjoelby et al., 1994). The basement membrane also becomes discontinuous and vanishes at the beginning of the dentin mineralization process (Kawashima and Okiji, 2016). Considering the critical roles of these key terms and the importance of fine balancing between complex networks of signaling pathways during the early odontogenic process, enriched terms may be involved in the pathogenesis of tooth agenesis.

Pathway-related enriched terms included “PI3K-Akt signaling pathway” and “α6β4 signaling pathway”. Several pathways act early in development to determine the differential location, identity, shape, and size of teeth. Even if no specific gene is responsible for each tooth shape, these enriched terms are more likely to be related to molar regions rather than incisor regions. BMP4 and FGF8 are the first molecular signals that initiate differential tooth morphogenesis (Laugel-Haushalter et al., 2013). *Fgf8* is expressed in the proximal (presumptive molar region) oral epithelium and promotes *Barx1* expression, whereas *Bmp4* is initially expressed in the distal (presumptive incisor) epithelium (Tucker et al., 1998). The PI3K-Akt signaling pathway has been associated with cell growth, cell cycle regulation, and cell survival. Recently, it has been proposed to function as an intracellular pathway for transducing FGF8 signals into nuclei in dental mesenchyme, preventing cell apoptosis (Lin et al., 2022). Integrin α6β4 is thought to mediate laminin-10/11-induced cell spreading and filopodia formation of the dental epithelium, implying that these interactions play an important role in determining the size and shape of tooth germs. The interaction between laminin-10/11 and integrin α6β4 is also thought to be required for PI3K/Akt pathway activation in the dental epithelium (Fukumoto et al., 2006). Furthermore, integrin α6β4 stimulates Rac1 and RhoA activation, which modulate cuspal shape decisions by coordinating adhesion junctions, actin distribution, and fibronectin localization to trigger inner dental epithelium invagination (Stewart and O’Connor, 2015; Li et al., 2016).

*PITX1*, which encodes a novel bicoid-related family of homeoproteins that is differentially expressed between the upper and lower molars, is one of the prime candidate genes for controlling maxillary/mandibular tooth identity (Laugel-Haushalter et al., 2013). *Pitx1* is expressed in the proximal mesenchyme of the developing mandible, hindlimb, oral epithelium, developing teeth, and pituitary gland. Inactivation of the *Pitx1* gene in mice impacts mandibular tooth morphogenesis (Mitsiadis and Drouin, 2008). Top-ranked terms from functional analyses of *Pitx1*-dependent genes have included the extracellular matrix during hindlimb development, and differentially expressed gene analysis between control and PITX1-overexpressing osteosarcoma genes also categorized the extracellular matrix and PI3K/Akt in the top 20 KEGG pathways (Nemec et al., 2017; Wang et al., 2018; Zhang et al., 2023).

Laminin α3 (the LAMA3 protein) is an element of laminin-5 (α3β3γ2) that regulates epithelial cell anchoring and motility via the integrins α6β4 and α3β1, respectively (Fukumoto and Yamada, 2005). In an animal study, *Lama3*-targeted knockout mice showed abnormalities in ameloblast differentiation (Ryan et al., 1999). *LAMA3* mutations have also been previously reported in non-syndromic hypodontia patients (Dinckan et al., 2018). We also identified a stop-gain variant (c.4750C>T) in *LAMA3* in our patients. To our knowledge, this is the third report of the *LAMA3* mutation in non-syndromic tooth agenesis. The exact mechanism by which *LAMA3* mutations lead to hypodontia is not fully understood, but it is thought to be related to the role of laminin-5 in tooth bud development and the attachment of teeth to the surrounding tissues. Further, other candidate genes sharing similar functions and enriched terms to *LAMA3* may also have a similar role in mandibular premolar hypodontia.

In a genome-wide association study (GWAS) of NSTA, including hypodontia and oligodontia, the rs917412-T variant was reported to be associated with the agenesis of mandibular second premolars (Jonsson et al., 2018). However, none of our samples had this variant possibly because of differences in allele frequency between different ethnicities. The allele frequency of rs917412-T is 0.0007704 in East Asians, which is significantly lower than the overall allele frequency of 0.2054 in the gnomAD database.

Regeneration of teeth is the most ideal treatment in dentistry and there are many advances such as tooth regenerative medicine strategies using targeted molecular therapy (Takahashi et al., 2020). But still many challenges are ahead such as the precise differentiation of dental stem cells into a specific tooth. Increased effort in expanding our comprehensive understanding through WES and bioinformatics is required for the future regenerative therapy.

There are some limitations to this pilot study. First, the sample size was small. Therefore, we attempted to overcome this shortcoming by specifying the phenotype of the disease. Second, the development of teeth is very complex, and interpretation of the impact variants have on the disease is challenging. Simple case-control comparisons may not be able to explain the pathogenicity of tooth agenesis. Pathways and signaling interactions that intersect and interplay during tooth development may be the key to the genotype–phenotype correlation. Third, we investigated genetic variants of unrelated individuals that share the same disease phenotype. Therefore, there is a need for segregation analysis to further verify candidate gene variant impact. Lastly, hypodontia may arise due to the mutations in non-coding regions such as gene regulatory domains. The application of whole-genome sequencing analysis may help further elucidate the pathogenesis of hypodontia.

In summary, we specified a phenotype for non-syndromic hypodontia as agenesis of the bilateral mandibular second premolars in a Korean population and attempted to reveal the pathogenesis of the disease using WES and bioinformatics. We found some known oligodontia gene variants in hypodontia patients, strengthening the possibility of additive effects in other genes and oligogenic inheritance in tooth agenesis. Furthermore, we identified some functionally enriched terms important to mandibular molar development and explored the possible common features of the etiology of mandibular premolar hypodontia. This study is a viable preliminary attempt to reveal the pathogenesis of tooth agenesis and may help design future tooth agenesis studies. Since there are various types of teeth, further studies with other phenotypes and larger populations are required to compare and understand the disease in the future.

## Data Availability

The datasets presented in this study can be found in online repositories. The names of the repository/repositories and accession number(s) can be found below: https://www.ncbi.nlm.nih.gov/, PRJNA983431.
